# The association between urgency level and hospital admission, mortality and resource utilization in three emergency department triage systems: an observational multicenter study

**DOI:** 10.1186/s13049-025-01392-5

**Published:** 2025-05-01

**Authors:** Marit E. van Wegen, Laura F. C. Fransen, Wendy A. M. H. Thijssen, Georgios Alexandridis, Bas de Groot

**Affiliations:** 1 Department of Emergency Medicine, Rijnstate Hospital, Arnhem, The Netherlands; 2 Department of Emergency Medicine, Canisius Wilhelmina Hospital, Nijmegen, The Netherlands; 3 Department of Emergency Medicine, Catharina Hospital, Eindhoven, The Netherlands; 4 Department of Emergency Medicine, Radboudumc, Nijmegen, The Netherlands; 5 Research Centre for Emergency Medicine, Aarhus University Hospital, Aarhus, Denmark

**Keywords:** Triage, Urgency level, Emergency medical services, Undertriage, Overtriage, In-hospital mortality, Hospitalization, Resource allocation

## Abstract

**Background:**

Effective triage systems are crucial for prioritizing patients based on urgency and optimizing resource utilization. An ideal triage system is expected to have low resource utilization, hospitalization and mortality among patients classified at low urgency levels. Furthermore, it should exhibit an increase in the risk of hospitalization and mortality as urgency levels increase, ensuring the most critically ill patients receive priority care first. However, it is unclear which triage system performs best.

**Objective:**

To compare the performance of the Manchester Triage System (MTS), the Emergency Severity Index (ESI), and the Netherlands Triage Standard (NTS) by investigating the association between urgency levels and resource utilization, hospitalization and in-hospital mortality in Emergency Department (ED) patients.

**Methods:**

Observational multicenter cohort study using data from the Netherlands Emergency department Evaluation Database, comprising seven representative EDs in six Dutch hospitals. All consecutive ED patients with a registered urgency level were included. Resource utilization, hospitalization and mortality were measured across all urgency levels. In each triage system, multivariable logistic regression was used to assess the association between urgency level and in-hospital mortality and hospitalization, adjusting for age, sex, presenting complaints and hospital type.

**Results:**

A total of 696,518 ED visits (MTS 320,406 (46.1%), ESI 214,267 (30.8%), NTS 161,845 (23.3%) patients) were included. Resource utilization was substantially lower in the lowest urgency level of the ESI compared to the MTS and NTS. Hospitalization to a regular ward, cardiac, medium or intensive care unit in the least urgent level was 3.9% in the ESI, considerably lower than in the MTS (23.1%) and NTS (34.3%) (*P* < 0.05). Mortality in the lowest urgency level of the ESI was 0.8%, while in the MTS and NTS this was 6.3% and 12.4%, respectively (*P* < 0.05). In the ESI, the risk (Adjusted Odds Ratios) for hospitalization and mortality increased much more with increasing urgency levels compared to the MTS and NTS.

**Conclusion:**

This study suggests that the ESI may be more effective in distinguishing between patients with low and high urgency, with a reduced risk of undertriage when compared to the MTS and NTS.

**Supplementary Information:**

The online version contains supplementary material available at 10.1186/s13049-025-01392-5.

## Introduction

Emergency department (ED) overcrowding is a global problem and a threat for patient safety, as well as the satisfaction of patients and healthcare providers [1–3]. There is a high and persistently increasing demand for emergency care while limited capacities and resources are available [1–6]. This higher demand on EDs may lead to poorer patient outcomes and quality of care [1–3, 6]. Adequate prioritization of ED patients using a triage system, in which patients are treated based on their urgency level and expected resources, is important and may increase patient safety [2, 3, 7, 8]. By categorizing patients into five or six different urgency levels, allowing patients with lower urgency to wait and ensuring more efficient allocation of time and resources to patients presenting with a higher urgency [8–11]. 

Despite the widespread use of various triage systems in clinical practice globally, multiple studies have demonstrated significant variability in the predictive performance of commonly implemented triage systems, such as the Emergency Severity Index (ESI) and the Manchester Triage System (MTS) [7, 8]. Additionally, several triage systems have been associated with considerable mistriage, either underestimating or overestimating the urgency of a patient’s condition, i.e. under- or overtriage [7, 8, 12]. It is unclear which triage system performs best in terms of predictive performance and mistriage due to a variety of outcome measures and study designs. More importantly, previous studies were often limited by a single center design or analysis of a single triage system, using small sample sizes, limited data or studying a specific population [7, 8, 13–15]. 

Currently, different triage systems are used in the Netherlands: The MTS, the ESI, the Netherlands Triage Standard (NTS) and local hospital triage tools [16]. Despite the usage of the NTS at several EDs in the Netherlands, little is known about the reliability, validity and performance of the NTS [17–20]. 

Comparison of the association between urgency levels and clinical outcomes in different triage systems will provide insight in which triage system is least affected by mistriage and has the best predictive performance. This information can be used for the improvement of current guidelines. Ideally, in a well performing triage system, a limited number of hospitalizations and no deaths would be expected in patients triaged as non-urgent. Furthermore, in the lower urgency levels, the number of used resources should be low [21]. Conversely, one expects the highest hospital admission and mortality rate in the highest urgency levels. Finally, there should be a clear association between the urgency level and the resources used, as well as mortality and hospitalization rate, independent of patient characteristics like age, sex and presenting complaints.

Given the variability in the performance of triage systems, a direct comparison of their ability to predict hospital admission, in-hospital mortality, and resource utilization is essential. Therefore, this study aims to investigate the association between urgency levels and hospital admission, in-hospital mortality and resource utilization in ED patients among three regularly used triage systems.

## Methods

### Study design and setting

An observational multicenter cohort study was conducted using data from the Netherlands Emergency department Evaluation Database (NEED). The NEED is a quality registry of EDs in the Netherlands providing insight in the quality of national ED care (www.stichting-need.nl). A detailed description of the data collected within the NEED is available in previously published studies utilizing this database [22, 23]. During the study period, the NEED existed of seven EDs in the Netherlands in six hospitals; two academic and four general hospitals [24]. The Medical Ethics Review Committee at Radboudumc determined that this study was exempt from the Medical Research Act and waived the need for informed consent (file no. 2023–16756).

### Study population

All consecutive ED patients with a registered urgency level were included in this study.

### Definitions

#### MTS: Manchester triage system

The MTS is a five-level ED triage system which assigns an urgency level based on the patient’s signs and symptoms. It consists of a reductive system using 53 different flowcharts [10]. Ruling out high priority signs or symptoms is necessary to reduce the patient’s urgency level. The urgency levels are divided into red (immediate), orange (very urgent), yellow (urgent), green (standard), blue (non-urgent) [10]. 

#### ESI: emergency severity index

The ESI is an ED triage system assigning priority using an algorithm based on the patient’s stability, vital signs and expected resources, resulting in an urgency level from one (most urgent) to five (least urgent) [9]. 

#### NTS: Netherlands triage standard

The NTS is a Dutch triage standard used by EDs, general practice centers and ambulance control rooms. It is a six-level system describing urgency levels from U0 (resuscitation) to U5 (no risk of harm, next workday) based on the patient’s condition [11]. 

### Data collection

Data from six hospitals was collected between January 1st, 2017 and December 31st, 2022. Hospital characteristics are displayed in Table 1. Resource utilization data, including blood tests, radiology, electrocardiogram (ECG) and interventions, were automatically extracted from hospital information systems via an Application Programming Interface (IPA) and transferred to the NEED. For example, any recorded blood test (i.e. hemoglobin, troponin, leukocytes) was registered as a resource utilization event.

**Table 1:** Hospital characteristics

Hospital	Academic/general	Triage system	Number of EDs	Period
1	Academic	NTS	1	January 1st, 2017 – September 2nd, 2018
		MTS		September 3rd, 2018 – December 31st, 2022
2	General	NTS	1	January 1st, 2017 – August 29th, 2018
		MTS		August 30th, 2018 – December 31st, 2022
3	Academic	MTS	1	January 1st, 2017 – June 8th, 2019
4	General	MTS	1	January 1st, 2020 – December 31st, 2022
5	General	NTS	1	January 1st, 2019 – December 1st, 2022
6	General	ESI	2	April 1st, 2018 – November 30th, 2022

**Legend**: MTS: Manchester Triage system; ESI: Emergency Severity Index; NTS: Netherlands Triage Standard; ED: Emergency Department

Participants within the different triage systems were stratified into four urgency levels: ‘not urgent’, ‘urgent’, ‘very urgent’ and ‘most urgent’. The NTS originally comprises six levels, wherein the lowest urgency levels of the NTS (‘negligible risk of harm, within 24 hours’ and ‘no risk of harm, next workday‘) were already combined in the database into a single category labelled ‘can be seen next day’. In addition, in the current database the two lowest urgency levels across all systems were merged into the ‘not urgent’ category, as the lowest level (‘can be seen next day’) included a small group of patients. Presenting complaints according to MTS, ESI and NTS were merged into one variable of synchronized presenting complaints (Additional File 1).

### Outcome measures

The primary outcomes were in-hospital mortality, hospital admission and resource utilization. In-hospital mortality was defined as death during hospital admission. Patients who died at the ED were not defined as in-hospital deaths but considered a separate category. Patients who died before arrival at the ED were excluded from analyses. Hospitalization included admission to a regular ward, medium care unit (MCU), intensive care unit (ICU), cardiac care unit (CCU) or transfer to another hospital. Resource utilization included the use of diagnostic tests, consultations and provided treatment. Secondary outcomes were length of ED stay, length of hospital stay and ED-revisit.

### Statistical analysis

#### Sample size calculation

Using the rule of thumb, at least ten events per potential confounder are necessary to avoid overfitting. To adjust for the thirteen variables, a minimum of 130 patients who died or were hospitalized was required. The NEED exceeded this threshold.

#### Descriptive statistics

Skewed data were presented as median with interquartile range. Categorical data were presented as number with percentages.

#### Main statistical analysis

Multivariable binary logistic regression analysis was used to assess the association between urgency level and in-hospital mortality and hospital admission in the three different triage systems. The primary association of interest was adjusted for age, sex, top ten presenting complaints and hospital type (academic versus general hospital). In all analyses, ‘not urgent’ was defined as the reference group. The reference category for hospital type was ‘general’ and ‘other’ for the top-10 presenting complaints. Patients who died before or upon arrival at the ED were excluded from the regression analysis.

Robustness was tested by adding and removing covariates, thereby ensuring the consistency on the association between triage category and outcomes. Multicollinearity was assessed by examining Variance Inflation Factors (VIFs). Adjusted odds ratios (AORs) were reported with 95% confidence intervals. A P-value < 0.05 was considered to be statistically significant.

Patients with missing data were excluded from the analyses. Data were analyzed using IBM SPSS Statistics (version: 29.0.0.0.).

## Results

### Patient inclusion and characteristics

The patient flowchart is shown in Additional File 2. A total of 696,518 ED visits were included for analysis. Patient characteristics are presented in Table 2, with additional characteristics presented in Additional File 3. The total MTS cohort comprised 320,406 (46.1%) patients, the ESI 214,267 (30.8%) patients and the NTS 161,845 (23.3%) patients. The median age of the cohort was 56 years old, with a majority of the patients being male (52.0%). The median age increased with higher urgency levels across all triage systems. The proportion of patients classified with the lowest urgency level was relatively low in the ESI cohort (12.9%), compared to the MTS (29.2%) and NTS (39.0%) cohorts. The percentage of patients marked with the highest urgency level was significantly higher in the NTS group (5.4%) than in the MTS (1.5%) and ESI (1.0%) groups.

**Table 2:** Patient characteristics in different triage systems and urgency levels

	Urgency level	Cohort	MTS	ESI	NTS
N(%)	Total	696518 (100)	320406 (100)	214267 (100)	161845 (100)
	Not urgent	184213 (26,4)	93456 (29,2)	27718 (12,9)	63039 (39,0)
	Urgent	364895 (52,4)	160377 (50,1)	147289 (68,7)	57229 (35,4)
	Very urgent	131749 (18,9)	61747 (19,3)	37117 (17,3)	32885 (20,3)
	Most urgent	15661 (2,2)	4826 (1,5)	2143 (1,0)	8692 (5,4)
**Demographics**	Total	56,0 [30,0–73,0]	56,0 [29–72]	57,0 [32–73]	56,0 [30–73]
Age, Median [IQR]	Not urgent	46,0 [21,0–68,0]	46,0 [20–68]	36,0 [19–60]	50,0 [25–70]
	Urgent	58,0 [33,0–74,0]	59,0 [34–73]	58,0 [35–74]	57,0 [30–74]
	Very urgent	61,0 [39,0–74,0]	60,0 [36–74]	62,0 [42–75]	62,0 [42–74]
	Most urgent	62,0 [45,0–74,0]	63,0 [42–74]	61,0 [42–74]	62,0 [46–74]
Sex (male), N (%)	Total	362257 (52,0)	165703 (51,7)	112502 (52,5)	84052 (51,9)
	Not urgent	97219 (52,8)	48787 (52,2)	16153 (58,3)	32279 (51,2)
	Urgent	185168 (50,7)	81492 (50,8)	74558 (50,6)	29118 (50,9)
	Very urgent	70628 (53,6)	32586 (52,8)	20451 (55,1)	17591 (53,5)
	Most urgent	9242 (59,1)	2838 (59,0)	1340 (62,5)	5064 (58,3)
**Mode of transport N (%)**	Total	662976 (95,2)	303242 (94,6)	206416 (96,3)	153318 (94,7)
	Missing	33542 (4,8)	17164 (5,4)	7851 (3,7)	8527 (5,3)
Ambulance	Total	229309 (100)	100449 (100)	78980 (100)	49880 (100)
	Not urgent	24704 (10,8)	12567 (12,5)	2260 (2,9)	9877 (19,8)
	Urgent	121949 (53,2)	50838 (50,6)	52465 (66,4)	18646 (37,4)
	Very urgent	70984 (31,0)	33068 (32,9)	22610 (28,6)	15306 (30,7)
	Most urgent	11672 (5,1)	3976 (4,0)	1645 (2,1)	6051 (12,1)
**Top ten presenting complaints** N (%)	Total	678840 (97,5)	308186 (96,2)	213390 (99,6)	156904 (97,2)
	Missing	17678 (2,5)	12220 (3,8)	877 (0,4)	4581 (2,8)
1. Extremity complaints	Total	142688 (100)	62746 (100)	46334 (100)	33608 (100)
	Not urgent	64457 (45,2)	35639 (56,8)	11293 (24,4)	17525 (52,1)
	Urgent	69144 (48,5)	24180 (38,5)	32210 (69,5)	12754 (37,9)
	Very urgent	8701 (6,1)	2906 (4,6)	2822 (6,1)	2973 (8,8)
	Most urgent	386 (0,3)	21 (0,0)	9 (0,0)	356 (1,1)
2. Feeling unwell	Total	113132 (100)	52053 (100)	30788 (100)	30291 (100)
	Not urgent	19938 (17,6)	10315 (19,8)	834 (2,7)	8789 (29,0)
	Urgent	59632 (52,7)	29050 (55,8)	19029 (61,8)	11553 (38,1)
	Very urgent	30758 (27,2)	11918 (22,9)	10662 (34,6)	8178 (27,0)
	Most urgent	2804 (2,5)	770 (1,5)	263 (0,9)	1771 (5,8)
3. Abdominal pain	Total	72587 (100)	33297 (100)	19742 (100)	19548 (100)
	Not urgent	16220 (22,3)	6610 (19,9)	469 (2,4)	9141 (46,8)
	Urgent	45096 (62,1)	21689 (65,1)	16220 (82,2)	7187 (36,8)
	Very urgent	10908 (15,0)	4872 (14,6)	3034 (15,4)	3002 (15,4)
	Most urgent	363 (0,5)	126 (0,4)	19 (0,1)	218 (1,0)
4. Dyspnea	Total	61926 (100)	29812 (100)	17011 (100)	15103 (100)
	Not urgent	9000 (14,5)	5597 (18,8)	176 (1,0)	3227 (21,4)
	Urgent	30905 (49,9)	14514 (48,7)	10398 (61,1)	5993 (39,7)
	Very urgent	20141 (32,5)	9060 (30,4)	6253 (36,8)	4828 (32,0)
	Most urgent	1880 (3,0)	641 (2,2)	184 (1,1)	1055 (7,0)
5. Chest pain	Total	53044 (100)	21252 (100)	22094 (100)	9698 (100)
	Not urgent	3598 (6,8)	2170 (10,2)	191 (0,9)	1237 (12,8)
	Urgent	34804 (65,6)	11334 (53,3)	20242 (91,6)	3228 (33,3)
	Very urgent	12615 (23,8)	7334 (34,5)	1620 (7,3)	3661 (37,8)
	Most urgent	2027 (3,8)	414 (1,9)	41 (0,2)	1572 (16,2)
6. Trauma (major)	Total	33534 (100)	14771 (100)	12379 (100)	6384 (100)
	Not urgent	5577 (16,6)	2020 (13,7)	1284 (10,4)	2273 (35,6)
	Urgent	15563 (46,4)	6215 (42,1)	6891 (55,7)	2457 (38,5)
	Very urgent	10010 (29,9)	5683 (38,5)	3396 (27,4)	940 (14,7)
	Most urgent	2375 (7,1)	853 (5,8)	808 (6,5)	714 (11,2)
7. Wounds	Total	27390 (100)	13028 (100)	5563 (100)	8799 (100)
	Not urgent	17461 (63,7)	8236 (63,2)	3117 (56,0)	6108 (69,4)
	Urgent	8272 (30,2)	4311 (33,1)	2245 (40,4)	1716 (19,5)
	Very urgent	1573 (5,7)	453 (3,5)	197 (3,5)	923 (10,5)
	Most urgent	84 (0,3)	28 (0,2)	4 (0,1)	52 (0,6)
8. Urinary problems	Total	16453 (100)	6407 (100)	5801 (100)	4245 (100)
	Not urgent	5471 (33,3)	1553 (24,2)	1398 (24,1)	2520 (59,4)
	Urgent	9026 (54,9)	4302 (67,1)	3352 (57,8)	1372 (32,3)
	Very urgent	1951 (11,9)	549 (8,6)	1051 (18,1)	351 (8,3)
	Most urgent	5 (0,0)	3 (0,0)	0 (0,0)	2 (0,0)
9. Falls	Total	15503 (100)	10009 (100)	5494 (100)	0 (0,0)
	Not urgent	4751 (30,6)	4260 (42,6)	491 (8,9)	0 (0,0)
	Urgent	8967 (57,8)	4887 (48,8)	4080 (74,3)	0 (0,0)
	Very urgent	1751 (11,3)	852 (8,5)	899 (16,4)	0 (0,0)
	Most urgent	34 (0,2)	10 (0,1)	24 (0,4)	0 (0,0)
10. Headache	Total	15144 (100)	9950 (100)	2892 (100)	2302 (100)
	Not urgent	2218 (14,6)	1075 (10,8)	192 (6,6)	951 (41,3)
	Urgent	7820 (51,6)	5123 (51,5)	2015 (69,7)	682 (29,6)
	Very urgent	4642 (30,7)	3558 (35,8)	676 (23,4)	408 (17,7)
	Most urgent	464 (3,1)	194 (1,9)	9 (0,3)	261 (11,3)

**Legend**: Values are median [IQR, interquartile range] or absolute number (percentage). MTS: Manchester Triage System; ESI: Emergency Severity Index; NTS: Netherlands Triage Standard; ED: Emergency Department; LOS: Length of stay. Top ten presenting complaints: based on top ten presenting complaints of the entire database

Approximately one-third of the patients arrived at the ED by ambulance, with most being referred by the general practitioner. Notably, a significant proportion of patients in the ‘not urgent’ group of the NTS were referred by a hospital specialist (51.6%). In contrast, the ‘most urgent’ group of the NTS had a relatively higher percentage of referrals from general practitioners (3.5%) compared to the MTS (0.4%) and ESI (0.3%).

### Resource utilization

Resource utilization and time spent in the ED is displayed in Table 3. Relative resource utilization, defined as resource use within a specific urgency level, divided by the total number of patients in that urgency level for the given triage system, is presented in Additional File 4. In the lowest urgency level of the ESI the fewest resources were utilized compared to the other two triage systems. In contrast, overall, the NTS had the highest resource utilization in both the lowest and highest urgency levels, while showing relatively lower resource use in the intermediate (‘urgent’) level compared to the MTS and ESI. The median ED length of stay (LOS) for the entire cohort was 2.7 h [1.8–3.8], with the shortest LOS observed among patients triaged as most or least urgent across all triage systems.

**Table 3:** Resources and time in the emergency department

		Cohort	MTS	ESI	NTS
**Diagnostics** N (%)					
Blood test	Missing	118 (0,0)	118 (0,0)	0 (0,0)	0 (0,0)
	Total	438,897 (100)	199,063 (100)	137,196 (100)	102,638 (100)
	Not urgent	64,925 (14,8)	31,667 (15,9)	3395 (2,5)	29,863 (29,1)
	Urgent	250,094 (57,0)	111,659 (56,1)	100,619 (73,3)	37,816 (36,8)
	Very urgent	109,933 (25,0)	51,524 (25,9)	31,290 (22,8)	27,119 (26,4)
	Most urgent	13,945 (3,2)	4213 (2,1)	1892 (1,4)	7840 (7,6)
Urine test (Sediment)	Missing	59,039 (8,5)	53,039 (18,4)	0 (0,0)	0 (0,0)
	Total	137,012 (100)	52,983 (100)	46,075 (100)	37,954 (100)
	Not urgent	21,131 (15,4)	7030 (13,3)	790 (1,7)	13,311 (35,1)
	Urgent	78,686 (57,4)	31,699 (59,8)	32,425 (70,4)	14,562 (38,4)
	Very urgent	34,015 (24,8)	13,087 (24,7)	12,672 (27,5)	8256 (21,8)
	Most urgent	3180 (2,3)	1167 (2,2)	188 (0,4)	1825 (4,8)
Radiology (Conventional, Ultrasound, CT)	Missing	220 (0,0)	105 (0,0)	0 (0,0)	115 (0,1)
	Total	408,286 (100)	188,836 (100)	128,969 (100)	90,481 (100)
	Not urgent	86,582 (21,2)	48,791 (25,8)	8955 (6,9)	28,836 (31,9)
	Urgent	219,461 (53,8)	95,999 (50,8)	88,506 (68,6)	34,956 (38,6)
	Very urgent	90,986 (22,3)	40,674 (21,5)	29,685 (23,0)	20,627 (22,8)
	Most urgent	11,257 (2,8)	3372 (1,8)	1823 (1,4)	6062 (6,7)
ECG	Missing	64,722 (9,3)	64,494 (20,1)	0 (0,0)	228 (0,1)
	Total	228,853 (100)	76,258 (100)	97,067 (100)	55,528 (100)
	Not urgent	21,721 (9,5)	10,429 (13,7)	1309 (1,3)	9983 (18,0)
	Urgent	134,180 (58,6)	43,813 (57,5)	69,788 (71,9)	20,579 (37,1)
	Very urgent	64,215 (28,1)	20,386 (26,7)	24,787 (25,5)	19,042 (34,3)
	Most urgent	8737 (3,8)	1630 (2,1)	1183 (1,2)	5924 (10,7)
**Interventions** N (%)					
Fluid administered	Missing	39,593 (5,7)	39,593 (12,4)	0 (0,0)	0 (0,0)
	Total	89,870 (100)	24,310 (100)	37,860 (100)	27,700 (100)
	Not urgent	10,966 (12,2)	1844 (7,6)	457 (1,2)	8665 (31,3)
	Urgent	44,911 (50,0)	12,006 (49,4)	20,885 (55,2)	12,020 (43,4)
	Very urgent	31,063 (34,6)	9674 (39,8)	15,454 (40,8)	5935 (21,4)
	Most urgent	2930 (3,3)	786 (3,2)	1064 (2,8)	1080 (3,9)
Medication administered	Missing	105 (0,0)	105 (0,0)	0 (0,0)	0 (0,0)
	Total	221,869 (100)	67,184 (100)	96,533 (100)	58,152 (100)
	Not urgent	32,391 (14,6)	8600 (12,8)	6558 (6,8)	17,233 (29,6)
	Urgent	122,008 (55,0)	35,976 (53,5)	62,994 (65,3)	23,038 (39,6)
	Very urgent	60,506 (27,3)	20,898 (31,1)	25,904 (26,8)	13,704 (23,6)
	Most urgent	6964 (3,1)	1710 (2,5)	1077 (1,1)	4177 (7,2)
**Consultations** N (%)	Total	689,661 (99,0)	313,549 (97,9)	214,267 (100)	161,845
	Missing	6857 (1,0)	6857 (2,1)	0 (0,0)	0 (0,0)
0	Total	390,193 (100)	123,598 (100)	180,919 (100)	85,676 (100)
	Not urgent	112,322 (28,8)	41,745 (33,8)	25,913 (14,3)	44,664 (52,1)
	Urgent	213,687 (54,8)	58,352 (47,2)	126,453 (69,9)	28,882 (33,7)
	Very urgent	60,521 (15,5)	22,306 (18,0)	27,666 (15,3)	10,549 (12,3)
	Most urgent	3663 (0,9)	1195 (1,0)	887 (0,5)	1581 (1,8)
1	Total	207,876 (100)	128,097 (100)	29,247 (100)	50,532 (100)
	Not urgent	50,409 (24,2)	35,207 (27,5)	1683 (5,8)	13,519 (26,8)
	Urgent	106,102 (51,0)	68,664 (53,6)	18,702 (63,9)	18,736 (37,1)
	Very urgent	44,681 (21,5)	22,430 (17,5)	8025 (27,4)	14,226 (28,2)
	Most urgent	6684 (3,2)	1796 (1,4)	837 (2,9)	4051 (8,0)
≥ 2	Total	91,592 (100)	61,854 (100)	4101 (100)	25,637 (100)
	Not urgent	16,943 (18,5)	11,965 (19,3)	122 (3,0)	4856 (18,9)
	Urgent	43,126 (47,1)	31,381 (50,7)	2134 (52,0)	9611 (37,5)
	Very urgent	26,234 (28,6)	16,698 (27,0)	1426 (34,8)	8110 (31,6)
	Most urgent	5289 (5,8)	1810 (2,9)	419 (10,2)	3060 (11,9)
**ED LOS (Hours), Median [IQR]**	Total	2,7 [1,8 − 3,8]	2,6 [1,7 − 3,6]	2,8 [1,9 − 4,1]	2,8 [1,8 − 3,9]
	Not urgent	2,0 [1,2–3,1]	1,9 [1,1–2,9]	1,7 [1,1–2,6]	2,5 [1,5 − 3,7]
	Urgent	2,9 [2,0–4,0]	2,8 [2,0–3,8]	2,9 [2,0–4,2]	2,9 [2,0–4,0]
	Very urgent	3,0 [2,2–4,2]	2,9 [2,1–4,0]	3,3 [2,3–4,7]	3,0 [2,2–4,1]
	Most urgent	2,4 [1,6 − 3,5]	2,0 [1,3–3,2]	1,9 [1,2–3,0]	2,7 [1,9 − 3,7]

**Legend**: Values are median [IQR, interquartile range] or absolute number (percentage). MTS: Manchester Triage system; ESI: Emergency Severity Index; NTS: Netherlands Triage Standard; ED: Emergency Department; LOS: Length of stay; ECG: Electrocardiogram

### Hospital admission and mortality

**Fig. 1:**
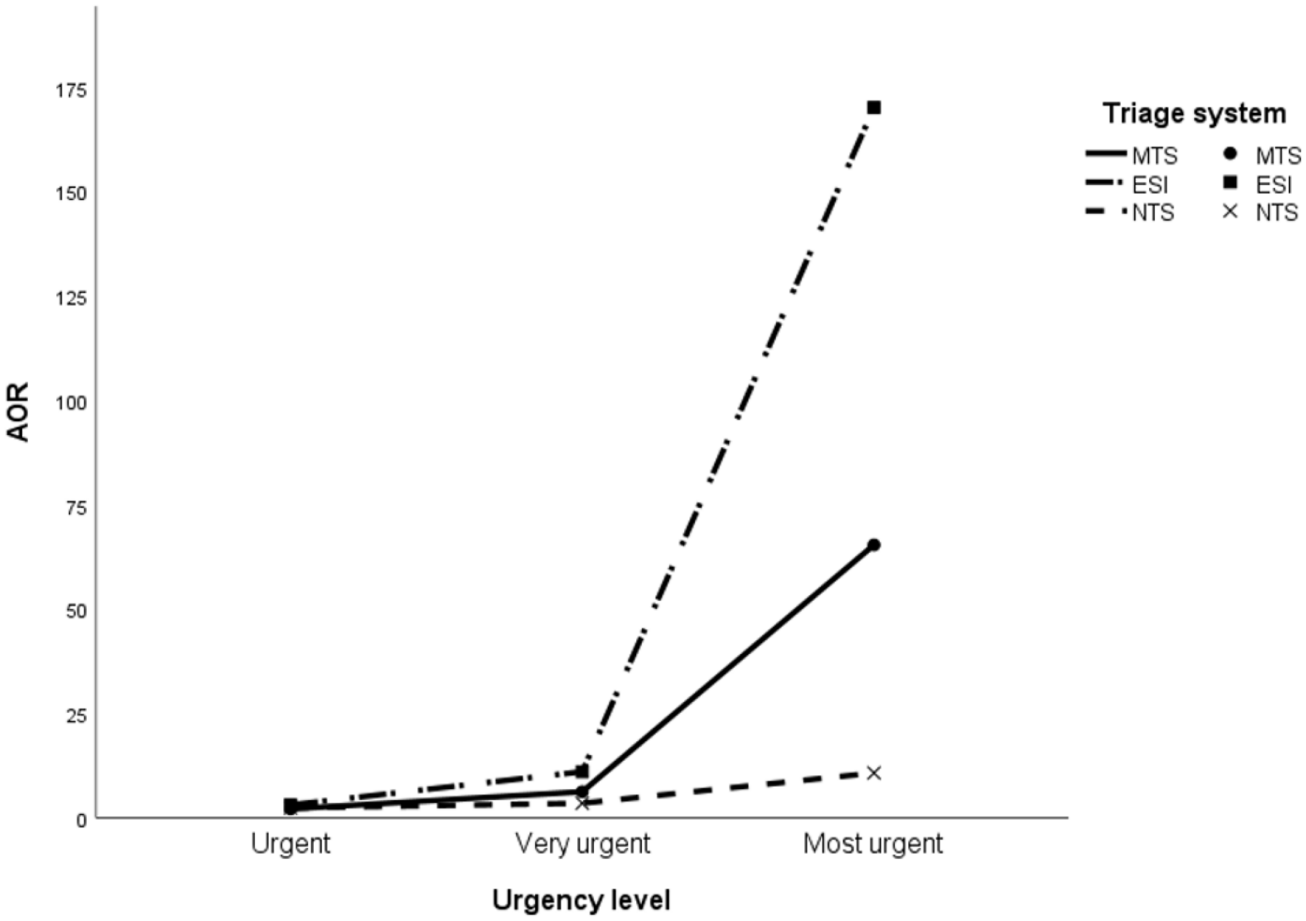
The association between urgency levels and in-hospital mortality. **Legend**: AOR: Adjusted Odds Ratio; MTS: Manchester Triage System; ESI: Emergency Severity Index; NTS: Netherlands Triage Standard. AOR adjusted for age, sex, top ten presenting complaints and hospital type (general (reference category) and academic). Reference group: not urgent. Top ten presenting complaints: (1) Extremity problems; (2) Feeling unwell; (3) Abdominal pain; (4) Dyspnea; (5) Chest pain; (6) Trauma major; (7) Wounds; (8) Urinary problems; (9) Falls; (10) Other (reference category)

**Fig. 2:**
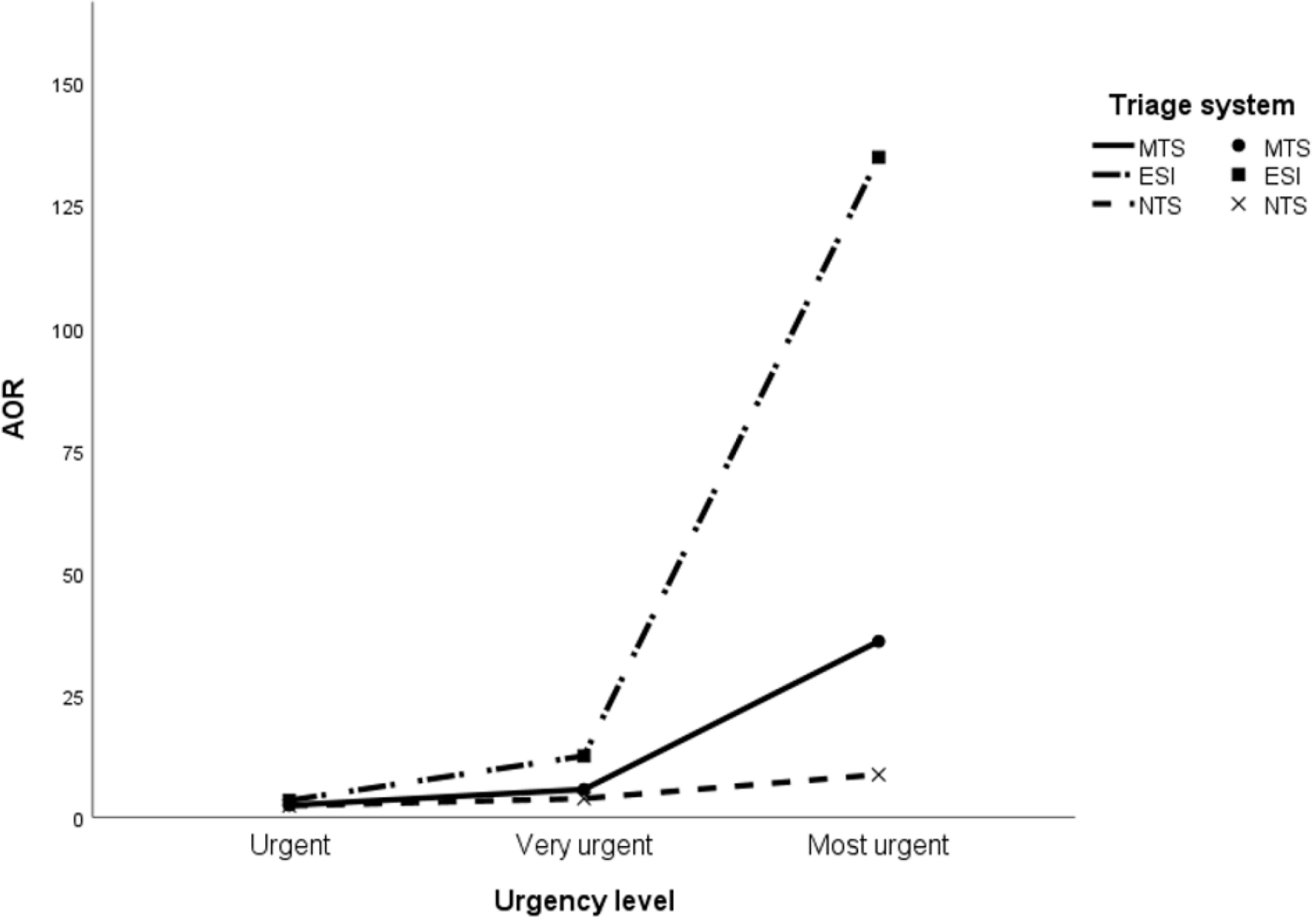
The association between urgency levels and hospital admission. **Legend**: AOR: Adjusted Odds Ratio; MTS: Manchester Triage System; ESI: Emergency Severity Index; NTS: Netherlands Triage Standard. AOR adjusted for age, sex, top ten presenting complaints and hospital type (general (reference category) and academic). Reference group: not urgent. Top ten presenting complaints: (1) Extremity problems; (2) Feeling unwell; (3) Abdominal pain; (4) Dyspnea; (5) Chest pain; (6) Trauma major; (7) Wounds; (8) Urinary problems; (9) Falls; (10) Other (reference category)

Table 4 presents the clinical outcomes per urgency level for each triage system. A higher percentage of patients in the ‘most urgent’ level of the NTS cohort was discharged home (3.5%) compared to the MTS (0.3%) and ESI (0.1%). Additionally, there was a remarkably high percentage of in-hospital mortality in the least urgent category for the NTS (12.4%), when compared with the MTS (6.3%) and the ESI (0.8%). Furthermore, hospitalization and in-hospital mortality rates were lower in the lowest urgency level of the ESI compared to the MTS and NTS. The median hospital LOS in the total cohort was 3.0 days [1.0–7.0]. The MTS and ESI showed the longest hospital LOS in the ‘most urgent’ category, whereas the NTS had the longest hospital LOS in the intermediate urgency categories (‘urgent’ and ‘very urgent’). AORs for in-hospital mortality and hospital admission are displayed in Figs. 1 and 2 and Additional File 5. Robustness check showed that the association between urgency levels and outcomes remained consistent after adjusting for different covariates. VIF values were approximately one, indicating that multicollinearity was not a concern in the regression models. The risk (AORs) for in-hospital mortality and hospital admission increases with incrementing urgency levels in all triage systems. However, the ESI demonstrated the most pronounced increase in risk for in-hospital mortality and hospitalization with increasing urgency levels.

**Table 4:** Clinical outcomes of emergency department patients

		Cohort	MTS	ESI	NTS
**Disposition** N (%)	Total	686,627 (98,6)	312,771 (97,6)	214,222 (100)	159,634 (98,6)
	Missing	9891 (1,4)	7635 (2,4)	45 (0,0)	2211 (1,4)
Discharged home	Total	326,877 (100)	145,704 (100)	126,102 (100)	55,071 (100)
	Not urgent	100,438 (30,7)	54,529 (37,4)	23,580 (18,7)	22,329 (40,5)
	Urgent	182,974 (56,0)	71,569 (49,1)	91,063 (72,2)	20,342 (36,9)
	Very urgent	41,009 (12,5)	19,203 (13,2)	11,334 (9,0)	10,472 (19,0)
	Most urgent	2456 (0,8)	403 (0,3)	125 (0,1)	1928 (3,5)
Regular ward	Total	248,461 (100)	117,715 (100)	68,007 (100)	62,739 (100)
	Not urgent	35,323 (14,2)	17,181 (14,6)	1826 (2,7)	16,316 (26,0)
	Urgent	136,496 (54,9)	66,869 (56,8)	44,601 (65,6)	25,026 (39,9)
	Very urgent	70,446 (28,4)	32,121 (27,3)	21,122 (31,1)	17,203 (27,4)
	Most urgent	6196 (2,5)	1544 (1,3)	458 (0,7)	4194 (6,7)
MCU/CCU	Total	13,098 (100)	5747 (100)	3903 (100)	3448 (100)
	Not urgent	585 (4,5)	398 (6,9)	22 (0,6)	165 (4,8)
	Urgent	5859 (44,7)	2345 (40,8)	2569 (65,8)	945 (27,4)
	Very urgent	5483 (41,9)	2738 (47,6)	1222 (31,3)	1523 (44,2)
	Most urgent	1171 (8,9)	266 (4,6)	90 (2,3)	815 (23,6)
ICU	Total	11,110 (100)	4413 (100)	4473 (100)	2224 (100)
	Not urgent	178 (1,6)	72 (1,6)	28 (0,6)	78 (3,5)
	Urgent	1751 (15,8)	619 (14,0)	729 (16,3)	403 (18,1)
	Very urgent	5401 (48,6)	2073 (47,0)	2482 (55,5)	846 (38,0)
	Most urgent	3780 (34,0)	1649 (37,4)	1234 (27,6)	897 (40,3)
Transfer to other hospital	Total	8224 (100)	5671 (100)	310 (100)	2243 (100)
	Not urgent	1206 (14,7)	679 (12,0)	34 (11,0)	493 (22,0)
	Urgent	3413 (41,5)	2433 (42,9)	173 (55,8)	807 (36,0)
	Very urgent	3015 (36,7)	2284 (40,3)	82 (26,5)	649 (28,9)
	Most urgent	590 (7,2)	275 (4,8)	21 (6,8)	294 (13,1)
Discharge against medical advice	Total	775 (100)	204 (100)	318 (100)	253 (100)
	Not urgent	344 (44,4)	96 (47,1)	112 (35,2)	136 (53,8)
	Urgent	336 (43,4)	81 (39,7)	176 (55,3)	79 (31,2)
	Very urgent	91 (11,7)	27 (13,2)	30 (9,4)	34 (13,4)
	Most urgent	4 (0,5)	0 (0,0)	0 (0,0)	4 (1,6)
Outpatient follow-up	Total	76,526 (100)	32,489 (100)	10,818 (100)	33,219 (100)
	Not urgent	40,113 (52,4)	15,583 (48,0)	2113 (19,5)	22,417 (67,5)
	Urgent	30,918 (40,4)	14,108 (43,4)	7938 (73,4)	8872 (26,7)
	Very urgent	5365 (7,0)	2783 (8,6)	756 (7,0)	1826 (5,5)
	Most urgent	130 (0,2)	15 (0,0)	11 (0,1)	104 (0,3)
General practice center	Total	105 (100)	56 (100)	0 (0,0)	49 (100)
	Not urgent	81 (77,1)	46 (82,1)	0 (0,0)	35 (71,4)
	Urgent	17 (16,2)	8 (14,3)	0 (0,0)	9 (18,4)
	Very urgent	7 (6,7)	2 (3,6)	0 (0,0)	5 (10,2)
	Most urgent	0 (0,0)	0 (0,0)	0 (0,0)	0 (0,0)
Deceased at ED	Total	1451 (100)	772 (100)	291 (100)	388 (100)
	Not urgent	16 (1,1)	9 (1,2)	2 (0,7)	5 (1,3)
	Urgent	82 (5,7)	40 (5,2)	31 (10,7)	11 (2,8)
	Very urgent	211 (14,5)	111 (14,4)	71 (24,4)	29 (7,5)
	Most urgent	1142 (78,7)	612 (79,3)	187 (64,3)	343 (88,4)
**In-hospital mortality N (%)**	Total	693,101 (99,5)	318,740 (99,5)	214,260 (100)	160,101 (98,9)
	Missing	3417 (0,5)	1666 (0,5)	7 (0,0)	1744 (1,1)
Deceased in hospital	Total	12,845 (100)	5529 (100)	4029 (100)	3287 (100)
	Not urgent	788 (6,1)	349 (6,3)	32 (0,8)	407 (12,4)
	Urgent	4426 (34,5)	1843 (33,3)	1500 (37,2)	1083 (32,9)
	Very urgent	5272 (41,0)	2254 (40,8)	1946 (48,3)	1072 (32,6)
	Most urgent	2359 (18,4)	1083 (19,6)	551 (13,7)	725 (22,1)
**7-day ED revisit** N (%)	Total	69,692 (100)	320,406 (100)	214,267 (100)	161,619 (99,9)
	Missing	226 (0,0)	0 (0,0)	0 (0,0)	226 (0,1)
Revisit with possible/ no relation to prior visit	Total	20,288 (100)	7908 (100)	5134 (100)	7246 (100)
	Not urgent	6563 (32,3)	2386 (30,2)	633 (12,3)	3544 (48,9)
	Urgent	10,170 (50,1)	4051 (51,2)	3625 (70,6)	2494 (34,4)
	Very urgent	3295 (16,2)	1406 (17,8)	862 (16,8)	1027 (14,2)
	Most urgent	260 (1,3)	65 (0,8)	14 (0,3)	181 (2,5)
Revisit with obvious relation to prior visit	Total	14,439 (100)	7711 (100)	4847 (100)	1881 (100)
	Not urgent	3038 (21,0)	2234 (29,0)	564 (11,6)	240 (12,8)
	Urgent	8437 (58,4)	4197 (54,4)	3513 (72,5)	727 (38,6)
	Very urgent	2789 (19,3)	1253 (16,2)	762 (15,7)	774 (41,1)
	Most urgent	175 (1,2)	27 (0,4)	8 (0,2)	140 (7,4)
**Hospital LOS (days), Median [IQR]**	Total	3,0 [1,0–7,0]	3,0 [1,0–6,0]	4,0 [2,0–7,0]	3,0 [1,0–7,0]
	Not urgent	3,0 [1,0–6,0]	3,0 [1,0–6,0]	2,0 [1,0–5,0]	3,0 [1,0–7,0]
	Urgent	3,0 [1,0–7,0]	3,0 [1,0–6,0]	3,0 [2,0–7,0]	4,0 [2,0–7,0]
	Very urgent	3,0 [1,0–7,0]	3,0 [1,0–7,0]	4,0 [2,0–8,0]	4,0 [1,0–7,0]
	Most urgent	4,0 [1,0–10,0]	4,0 [1,0–11,0]	5,0 [1,0–13,0]	3,0 [1,0–8,0]

Legend: Values are median [IQR, interquartile range] or absolute number (percentage). MTS: Manchester Triage system; ESI: Emergency Severity Index; NTS: Netherlands Triage Standard; ED: Emergency Department; MCU: Medium Care Unit; CCU: Cardiac Care Unit; ICU: Intensive Care Unit; LOS: Length of stay

## Discussion

Previous studies have shown considerable variability in the performance of different triage systems in predicting patient outcomes such as hospital admission and mortality. This study suggests that the ESI may be more effective in distinguishing between patients with low and high urgency compared to the MTS and NTS. The substantial proportion of ED patients who are hospitalized and die in the low urgency levels suggest a higher risk of undertriage in the MTS and NTS, which may impact patient outcomes and resource allocation.

Previous systematic reviews assessing the performance of the ESI and MTS, conducted by Hinson et al. (15 studies on the ESI and 14 on the MTS) and Zachariasse et al. (21 studies on the ESI and 15 on the MTS), concluded that there is no clear preference for either system, as their overall performance appears comparable [7, 8]. However, many of these observational studies were limited by single-center designs, small or specific patient populations, and differences in settings and healthcare systems, limiting their generalizability [7, 8, 13, 25, 26]. Furthermore, direct head-to-head comparisons of the MTS and ESI within a single study and across large, diverse patient populations remain scarce [25, 26]. Our findings add to the existing evidence by demonstrating that the adjusted risk of hospital admission increased significantly more with increasing urgency levels in the ESI compared to the MTS and NTS, suggesting that urgency levels of the ESI better discriminate the need for hospitalization. This aligns with previous findings by van der Wulp et al. [25] Additionally, our study extends prior research by examining in-hospital mortality and adjusting for presenting complaints, factors that were not considered in earlier studies.

The few studies suggesting that the NTS is a valid triage system are limited by small sample sizes, single-center designs, or its reliance on case scenarios [19, 20]. These studies also report an elevated risk of both under- and overtriage in the NTS, a finding consistent with our results [19, 20]. By being the first multicenter study to comprehensively assess the performance of the NTS in ED triage and directly compare it to other triage systems, our study provides a broader and more generalizable perspective on its effectiveness.

The pattern of low resource utilization, hospitalization, and in-hospital mortality in the lowest urgency level and progressively higher levels in the higher urgency levels, suggests that the ESI more effectively differentiates between low- and high-acuity patients compared to the MTS and NTS. The ESI appears to be least affected by undertriage as it shows the lowest rates of resource utilization, hospital admissions, and in-hospital mortality in the lowest urgency level compared to the MTS and NTS, which exhibit a substantial risk for hospitalization and mortality in the lowest urgency level. Lower resource utilization in the lowest urgency levels of the ESI, compared to the MTS and NTS, would be expected as the ESI incorporates resource utilization into its triage algorithm. The anticipated need for multiple resources during initial assessment generally results in patients being assigned to at least an intermediate urgency level, or to a higher level if vital signs are abnormal [9]. Additionally, a higher proportion of patients were triaged as ‘urgent’ in the ESI (68.7%) compared to the MTS (50.1%) and NTS (35.4%). This suggests that the ESI system may be more effective in identifying patients who require more intensive resources for assessment, reflecting an appropriate triage process where additional resources are needed to determine whether a patient can be safely discharged.

Furthermore, the NTS possibly has an elevated risk of overtriage, as suggested by a greater proportion of patients discharged home from the highest urgency levels. This could result in greater strain on the ED, potentially depleting resources and affecting care for other patients.

There are indications that triage systems may perform differently across various age groups [14, 15, 27]. While our study evaluated the performance of triage systems across all age groups, future research should compare how these systems perform specifically within both pediatric and geriatric populations. Furthermore, as EDs experience increasing crowding, implementing a valid triage system is crucial, and adding additional triage methods could be beneficial. A simple triage score that incorporates mobility, mental status, and oxygen saturation has been shown to identify twice as many patients at low risk of early death compared to the ESI [28]. Enhancing the ESI by integrating measures of mobility and mental status into its protocol could potentially improve its performance.

This study has several limitations. First, a retrospective observational study is susceptible to potential documentation or data entry errors. However, as the data entry process was largely automated, the risk of misregistration was minimized. Second, the ESI triage system was used in only one hospital, which may affect the generalizability of the findings to other settings. Nonetheless, this hospital includes two ED locations and a large study population, which helps mitigate this concern. In addition, there could be potential case-mix differences between different hospital populations. To mitigate this, hospital type was included as a covariate in the multivariable logistic regression model, reducing the potential confounding effect of hospital type on the association between triage category and outcome. However, we believe that the case-mixes in terms of comorbidity and complexity are comparable between the hospitals. As shown in Table 2, patient characteristics such as age, sex, arrival by ambulance (a measure of disease severity), and presenting complaints are similar across hospitals. Additionally, Table 3 demonstrates that the proportion of patients undergoing blood tests, radiological tests, or interventions relative to the total number of patients in the ESI, MTS, and NTS groups is also comparable, further suggesting that case-mix differences are unlikely to explain the observed results.

Furthermore, the synchronization of presenting complaints of the MTS, ESI and NTS to enable comparison, may have introduced some categorization errors. Yet, no substantial differences were observed between the groups after merging the presenting complaints.

A key strength of this study is its multicenter design, which includes both academic and general hospitals across multiple locations. The large cohort size in this study further strengthens the generalizability of the findings. The use of a consistent study design and similar outcome measures to compare triage systems within the same healthcare system reduces the influence of external factors, such as inter-country differences and variations in healthcare systems, on triage performance. Furthermore, conducting a multivariable regression analysis, with adjustments for potential confounders such as age, gender, presenting complaint, and hospital type, enhances the reliability of our findings.

## Conclusion

The sharper increase in risk for hospital admission and mortality with increasing urgency level suggests that the ESI more effectively discriminates between low and high urgency levels. The substantial proportion of ED patients who are hospitalized and die in the low urgency levels suggest a higher risk of undertriage in the MTS and NTS. Future studies should explore performance differences between these triage systems across various age groups.

## Electronic supplementary material

Below is the link to the electronic supplementary material.

Additional File 1 Synchronization of presenting complaints

Additional File 2 Flowchart of patient inclusion

Additional File 3 Additional patient characteristics

Additional File 4 Relative resource utilization

Additional File 5 Odds ratios

## Data Availability

No datasets were generated or analysed during the current study.
