# Supplementary material for: The association between urgency level and hospital admission, mortality and resource utilization in three emergency department triage systems: an observational multicenter study
Source: Scand J Trauma Resusc Emerg Med. 2025 May 1;33:72. doi: 10.1186/s13049-025-01392-5 (PMC12044865; doi:10.1186/s13049-025-01392-5)
Supplement: Supplementary file 1 — Additional File 1 Synchronization of presenting complaints [file 13049_2025_1392_MOESM1_ESM.docx]

**Additional File 1**

**Synchronization of the presenting complaints in the MTS, NTS and ESI**

|  | | | |
| --- | --- | --- | --- |
| **Synchronized presenting complaints used in the study**  **(52 in total)** | **MTS presenting complaints**  **(51 in total)** | **NTS presenting complaints**  **(50 in total)** | **ESI presenting complaints**  **(83 in total)** |
| 62. Abdominal pain | 14. Abdominal pain in adults  13. Abdominal pain in children | 11. Abdominal pain in adults  10. Abdominal pain in children | 9. Abdominal pain  73. Suspected abdominal aneurysm |
| 2. Abscesses and local infections | 2. Abscesses and local infections |  | 82. Wound infection  84. Swelling |
| 61 Allergy, bites & stings | 5. Allergy  9. Bites and stings | 2. Allergic reaction and stings | 3. Allergic reaction  31 Itches |
| 36. Apparently drunk | 36. Apparently drunk |  |  |
| 34. Assault | 34. Assault |  | 20. Assault |
| 45. Back pain | 45. Back pain | 37. Back pain | 52. Back pain |
| 49. Behaving strangely or suicidal | 49. Behaving strangely | 47. Behaving strangely or suicidal | 74. Confused  76. Behaving strangely |
| 53. Breast infection |  | 7. Breast infection |  |
| 12. Burns | 12. Burns | 9. Burns | 8. Burns |
| 42. Chest pain | 42. Chest pain | 35. Thoracic pain | 56. Thoracic pain |
| 15. Collapse | 15. Collapse adult | 48. Collapse | 10. Collapse  50. Resuscitation  55. Return of spontaneous circulation (ROSC) |
| 56. Constipation |  | 31. Constipation |  |
| 54. Coughing |  | 19. Coughing |  |
| 28. Crying baby | 28. Crying baby |  | 26. Crying baby |
| 20. Dental problems | 20. Dental problems | 16. Dental problems | 46. Jaw pain |
| 17. Diabetes | 17. Diabetes | 13. Diabetes |  |
| 11. Diarrhea and vomiting | 11. Diarrhea and vomiting | 14. Diarrhea  8. Vomiting | 7. Vomiting  13. Diarrhea  39. Nausea |
| 63. Dyspnea | 32. Shortness of breath in adults  6. Asthma  31. Shortness of breath in children | 28. Shortness of breath | 16. Dyspnea |
| 38. Ear problems | 38. Ear problems | 33. Ear problems | 43. Ear problems |
| 10. Exposure to chemicals | 10. Exposure to chemicals |  | 28. Inhalation trauma |
| 18. Extremity problems | 18. Extremity problems | 3. Arm problems  39. General/limb trauma  4. Leg problems | 21. Cast problems  37. Luxation  47. Pain from extremity  57. Trauma arm  58. Trauma leg  59. Trauma pelvis/hip  65. Trauma wrist/hand  61. Trauma ankle/feet  75. Finger injury  5. Proven Deep Venous Thrombosis  71. Vascular problems |
| 37. Eye problems | 37. Eye problems | 32. Eye problems | 42. Eye problems |
| 1. Facial problems | 1 Facial problems | 38. Facial trauma  6. Nosebleed | 40. Nosebleed |
| 23. Falls | 23. Falls |  | 72. Falls |
| 60. Feeling unwell | 4. Unwell adult  3. Unwell child | 27. Fever in adults  26. Fever in children  30. Neurological failure  1. Unwell adult  50. Unwell child  15. Dizziness | 2. Feeling unwell  4. Amnesia  12. Dehydration  15 Dizziness  17. Decreased EMV-score  27. Icterus  33. Complaints after chemotherapy  35. Fever  36. Fever after chemotherapy  41. Low temperature  53. Sepsis  54. Shock  69. Neurological deficit |
| 16. Foreign body | 16. Foreign body | 12. Foreign body | 11. Foreign body |
| 19. (Gastro-intestinal) bleeding | 19. Gastro-intestinal (GI) bleeding |  | 6. Bleeding  18. Gastro-intestinal bleeding |
| 41. Genital problems | 41. Testicular pain | 17. Genital problems |  |
| 26. Headache | 26. Headache | 20. Headache | 24. Headache |
| 55. Implantable Cardioverter Defibrillator (ICD) |  | 22. Implantable Cardioverter Defibrillator (ICD) |  |
| 21. Irritable child | 21. Irritable child |  | 19. Irritable child |
| 33. Limping child | 33. Limping child |  |  |
| 24. Major incidents | 24. Major incidents |  | 49. Disaster |
| 64. Major trauma | 25. Head injury  46. Major trauma  44. Torso injury | 40. Abdominal trauma  42. Back trauma  43. Head trauma  44. Thorax trauma | 62. Head trauma  68. Thoracic trauma  60. Abdominal trauma  67. Back trauma  66. Trauma torso  63. Unstable trauma  23. Head injury  38. Multi trauma |
| 43. Mental illness | 43. Mental illness |  | 48. Psychiatric disorder |
| 52. Near-drowning |  | 5. Near-drowning |  |
| 35. Neck pain | 35. Neck pain | 29. Neck problems  41. Neck trauma | 64. Trauma neck |
| 39. Overdose and poisoning | 39. Overdose and poisoning | 24. Intoxication | 30. Intoxication |
| 40. Palpitations | 40. Palpitations | 18. Palpitations | 45. Palpitations |
| 48. Per vaginum bleeding | 48. Per vaginum bleeding | 46. Per vaginum bleeding |  |
| 51. Pregnancy/childbirth | 51. Pregnancy | 34. Childbirth |  |
| 27. Rashes | 27. Rash | 21. Skin problems | 25. Skin abnormalities |
| 57. Rectal problems |  | 36. Rectal problems |  |
| 29. Seizures | 29. Seizure | 23. Seizures | 29. Seizure/convulsion |
| 7. Self-harm | 7. Self-harm |  |  |
| 22. Sexually transmitted diseases | 22. Sexually transmitted diseases |  |  |
| 30. Throat problems | 30. Sore throat | 25. Throat problems | 32. Throat problems |
| 47. Urinary problems | 47. Urinary problems | 45. Urinary problems | 70. Urogenital problems  14. Drainage/Catheter problems |
| 8. Worried parent | 8. Worried parent |  |  |
| 50. Wounds | 50. Wounds | 49. Wound | 83. Wound torso  81. Wound head  79. Wound pelvis/hip  77. Wound arm  78. Wound leg  80. Abdominal wound |
| 85. Additional |  |  | 44. Transfer from other hospital  34. Complaints after medical intervention  22. Reassessment  1. Abnormal laboratory results |

**Legend:** MTS = Manchester Triage System; NTS = Netherlands Triage System; ESI = Emergency Severity Index, EMV score = Eye Motor Verbal-score
