# Supplementary figures and images for: The association between urgency level and hospital admission, mortality and resource utilization in three emergency department triage systems: an observational multicenter study
Source: Scand J Trauma Resusc Emerg Med. 2025 May 1;33:72. doi: 10.1186/s13049-025-01392-5 (PMC12044865; doi:10.1186/s13049-025-01392-5)

**Additional File 2: Flowchart of patient inclusion**

**
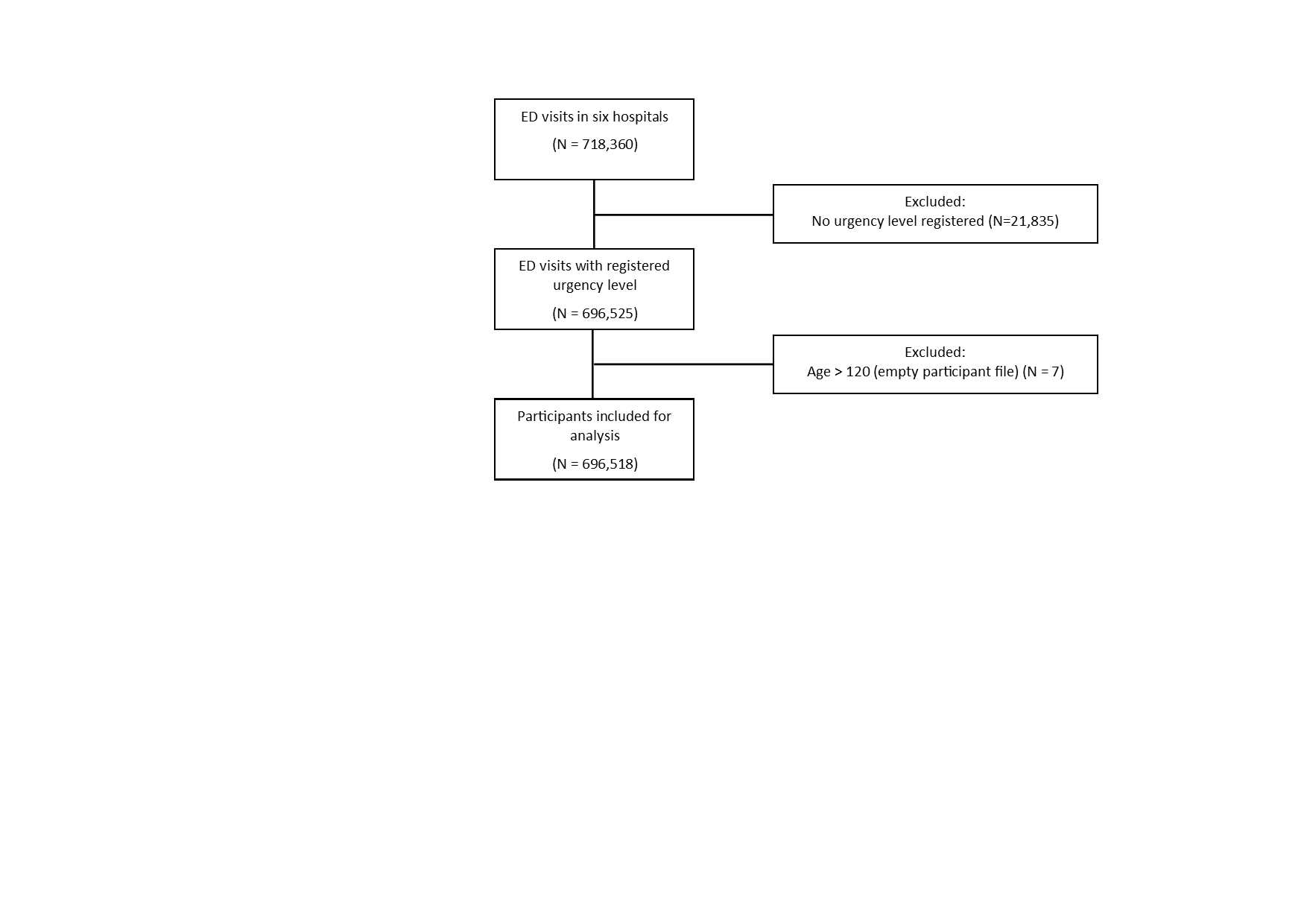
**

Supplement: Supplementary file 2 — Additional File 2 Flowchart of patient inclusion [file 13049_2025_1392_MOESM2_ESM.docx]
