# Supplementary material for: The association between urgency level and hospital admission, mortality and resource utilization in three emergency department triage systems: an observational multicenter study
Source: Scand J Trauma Resusc Emerg Med. 2025 May 1;33:72. doi: 10.1186/s13049-025-01392-5 (PMC12044865; doi:10.1186/s13049-025-01392-5)
Supplement: Supplementary file 3 — Additional File 3 Additional patient characteristics [file 13049_2025_1392_MOESM3_ESM.docx]

**Additional File 3: Additional patient characteristics**

Patient characteristics in different triage systems and urgency levels

|  | **Urgency level** | **Cohort** | **MTS** | **ESI** | **NTS** |
| --- | --- | --- | --- | --- | --- |
| N (%) | Total | 696518 (100) | 320406 (100) | 214267 (100) | 161845 (100) |
|  | Not urgent  Urgent  Very urgent  Most urgent | 184213 (26,4)  364895 (52,4)  131749 (18,9)  15661 (2,2) | 93456 (29,2)  160377 (50,1)  61747 (19,3)  4826 (1,5) | 27718 (12,9)  147289 (68,7)  37117 (17,3)  2143 (1,0) | 63039 (39,0)  57229 (35,4)  32885 (20,3)  8692 (5,4) |
| **Demographics** | Total | 56,0 [30,0-73,0] | 56,0 [29-72] | 57,0 [32-73] | 56,0 [30-73] |
| Age | Not urgent | 46,0 [21,0-68,0] | 46,0 [20-68] | 36,0 [19-60] | 50,0 [25-70] |
| Median [IQR] | Urgent | 58,0 [33,0-74,0] | 59,0 [34-73] | 58,0 [35-74] | 57,0 [30-74] |
|  | Very urgent | 61,0 [39,0-74,0] | 60,0 [36-74] | 62,0 [42-75] | 62,0 [42-74] |
|  | Most urgent | 62,0 [45,0-74,0] | 63,0 [42-74] | 61,0 [42-74] | 62,0 [46-74] |
|  |  |  |  |  |  |
| Sex (male) N (%) | Total | 362257 (52,0) | 165703 (51,7) | 112502 (52,5) | 84052 (51,9) |
|  | Not urgent | 97219 (52,8) | 48787 (52,2) | 16153 (58,3) | 32279 (51,2) |
|  | Urgent | 185168 (50,7) | 81492 (50,8) | 74558 (50,6) | 29118 (50,9) |
|  | Very urgent | 70628 (53,6) | 32586 (52,8) | 20451 (55,1) | 17591 (53,5) |
|  | Most urgent | 9242 (59,1) | 2838 (59,0) | 1340 (62,5) | 5064 (58,3) |
| **Mode of transport** | Total | 662976 (95,2) | 303242 (94,6) | 206416 (96,3) | 153318 (94,7) |
| N (%) | Missing | 33542 (4,8) | 17164 (5,4) | 7851 (3,7) | 8527 (5,3) |
| Ambulance | Total | 229309 (100) | 100449 (100) | 78980 (100) | 49880 (100) |
|  | Not urgent | 24704 (10,8) | 12567 (12,5) | 2260 (2,9) | 9877 (19,8) |
|  | Urgent | 121949 (53,2) | 50838 (50,6) | 52465 (66,4) | 18646 (37,4) |
|  | Very urgent | 70984 (31,0) | 33068 (32,9) | 22610 (28,6) | 15306 (30,7) |
|  | Most urgent | 11672 (5,1) | 3976 (4,0) | 1645 (2,1) | 6051 (12,1) |
| **Referral** N (%) | Total  Missing | 677710 (97,3)  18808 (2,7) | 319258 (99,6)  1148 (0,4) | 203114 (94,8)  11153 (5,2) | 155338 (96,0)  6507 (4,0) |
| Self-referral | Total | 230079 (100) | 103715 (100) | 71210 (100) | 55154 (100) |
|  | Not urgent | 56502 (24,6) | 24268 (23,4) | 13226 (18,6) | 19008 (34,5) |
|  | Urgent | 107539 (46,7) | 47129 (45,4) | 41382 (58,1) | 19028 (34,5) |
|  | Very urgent | 55855 (24,3) | 28580 (27,6) | 15331 (21,5) | 11944 (21,7) |
|  | Most urgent | 10183 (4,4) | 3738 (3,6) | 1271 (1,8) | 5174 (9,4) |
| General practitioner | Total | 367316 (100) | 169173 (100) | 123156 (100) | 74987 (100) |
|  | Not urgent | 95399 (26,0) | 55221 (32,6) | 12043 (9,8) | 28135 (37,5) |
|  | Urgent | 210727 (57,4) | 89451 (52,9) | 92731 (75,3) | 28545 (38,1) |
|  | Very urgent | 57450 (15,6) | 23827 (14,1) | 17972 (14,6) | 15651 (20,9) |
|  | Most urgent | 3740 (1,0) | 674 (0,4) | 410 (0,3) | 2656 (3,5) |
| Specialist | Total | 80315 (100) | 46370 (100) | 8748 (100) | 25197 (100) |
|  | Not urgent | 27901 (34,7) | 13710 (29,6) | 1181 (13,5) | 13010 (51,6) |
|  | Urgent | 36649 (45,6) | 23249 (50,1) | 6295 (72,0) | 7105 (28,2) |
|  | Very urgent | 14645 (18,2) | 9052 (19,5) | 1214 (13,9) | 4379 (17,4) |
|  | Most urgent | 1120 (1,4) | 359 (0,8) | 58 (0,7) | 703 (2,8) |

| **Location** N (%) | Total  Missing | 536849 (77,1)  159669 (22,9) | 190225 (59,4)  130181 (40,6) | 214267 (100)  0 (0,0) | 132357 (81,8)  29488 (18,2) | |
| --- | --- | --- | --- | --- | --- | --- |
| Shock/trauma room | Total | 45405 (100) | 18832 (100) | 17050 (100) | 9523 (100) | |
|  | Not urgent | 3933 (8,7) | 2381 (12,6) | 164 (1,0) | 1388 (14,6) | |
|  | Urgent | 17440 (38,4) | 8725 (46,3) | 5522 (32,4) | 3193 (33,5) | |
|  | Very urgent | 19199 (42,3) | 6332 (33,6) | 9848 (57,8) | 3019 (31,7) | |
|  | Most urgent | 4833 (10,6) | 1394 (7,4) | 1516 (8,9) | 1923 (20,2) | |
| **Top ten presenting complaints** N (%) | Total  Missing | 678840 (97,5)  17678 (2,5) | 308186 (96,2)  12220 (3,8) | 213390 (99,6)  877 (0,4) | 156904 (97,2)  4581 (2,8) | |
| 1. Extremity complaints | Total | 142688 (100) | 62746 (100) | 46334 (100) | 33608 (100) | |
|  | Not urgent | 64457 (45,2) | 35639 (56,8) | 11293 (24,4) | 17525 (52,1) | |
|  | Urgent | 69144 (48,5) | 24180 (38,5) | 32210 (69,5) | 12754 (37,9) | |
|  | Very urgent | 8701 (6,1) | 2906 (4,6) | 2822 (6,1) | 2973 (8,8) | |
|  | Most urgent | 386 (0,3) | 21 (0,0) | 9 (0,0) | 356 (1,1) | |
|  |  |  |  |  |  | |
| 1. Feeling unwell | Total | 113132 (100) | 52053 (100) | 30788 (100) | 30291 (100) | |
|  | Not urgent | 19938 (17,6) | 10315 (19,8) | 834 (2,7) | 8789 (29,0) | |
|  | Urgent | 59632 (52,7) | 29050 (55,8) | 19029 (61,8) | 11553 (38,1) | |
|  | Very urgent | 30758 (27,2) | 11918 (22,9) | 10662 (34,6) | 8178 (27,0) | |
|  | Most urgent | 2804 (2,5) | 770 (1,5) | 263 (0,9) | 1771 (5,8) | |
|  |  |  |  |  |  | |
| 1. Abdominal pain | Total | 72587 (100) | 33297 (100) | 19742 (100) | 19548 (100) | |
|  | Not urgent | 16220 (22,3) | 6610 (19,9) | 469 (2,4) | 9141 (46,8) | |
|  | Urgent | 45096 (62,1) | 21689 (65,1) | 16220 (82,2) | 7187 (36,8) | |
|  | Very urgent | 10908 (15,0) | 4872 (14,6) | 3034 (15,4) | 3002 (15,4) | |
|  | Most urgent | 363 (0,5) | 126 (0,4) | 19 (0,1) | 218 (1,0) | |
|  |  |  |  |  |  | |
| 1. Dyspnea | Total | 61926 (100) | 29812 (100) | 17011 (100) | 15103 (100) | |
|  | Not urgent | 9000 (14,5) | 5597 (18,8) | 176 (1,0) | 3227 (21,4) | |
|  | Urgent | 30905 (49,9) | 14514 (48,7) | 10398 (61,1) | 5993 (39,7) | |
|  | Very urgent | 20141 (32,5) | 9060 (30,4) | 6253 (36,8) | 4828 (32,0) | |
|  | Most urgent | 1880 (3,0) | 641 (2,2) | 184 (1,1) | 1055 (7,0) | |
|  |  |  |  |  |  | |
| 1. Chest pain | Total | 53044 (100) | 21252 (100) | 22094 (100) | 9698 (100) | |
|  | Not urgent | 3598 (6,8) | 2170 (10,2) | 191 (0,9) | 1237 (12,8) | |
|  | Urgent | 34804 (65,6) | 11334 (53,3) | 20242 (91,6) | 3228 (33,3) | |
|  | Very urgent | 12615 (23,8) | 7334 (34,5) | 1620 (7,3) | 3661 (37,8) | |
|  | Most urgent | 2027 (3,8) | 414 (1,9) | 41 (0,2) | 1572 (16,2) | |
|  |  |  |  |  |  | |
| 1. Trauma (major) | Total | 33534 (100) | 14771 (100) | 12379 (100) | 6384 (100) | |
|  | Not urgent | 5577 (16,6) | 2020 (13,7) | 1284 (10,4) | 2273 (35,6) | |
|  | Urgent | 15563 (46,4) | 6215 (42,1) | 6891 (55,7) | 2457 (38,5) | |
|  | Very urgent | 10010 (29,9) | 5683 (38,5) | 3396 (27,4) | 940 (14,7) | |
|  | Most urgent | 2375 (7,1) | 853 (5,8) | 808 (6,5) | 714 (11,2) | |
|  |  |  |  |  |  | |
| 1. Wounds | Total | 27390 (100) | 13028 (100) | 5563 (100) | 8799 (100) | |
|  | Not urgent | 17461 (63,7) | 8236 (63,2) | 3117 (56,0) | 6108 (69,4) | |
|  | Urgent | 8272 (30,2) | 4311 (33,1) | 2245 (40,4) | 1716 (19,5) | |
|  | Very urgent | 1573 (5,7) | 453 (3,5) | 197 (3,5) | 923 (10,5) | |
|  | Most urgent | 84 (0,3) | 28 (0,2) | 4 (0,1) | 52 (0,6) | |
|  |  |  |  |  |  | |
| 1. Urinary problems | Total | 16453 (100) | 6407 (100) | 5801 (100) | 4245 (100) | |
|  | Not urgent | 5471 (33,3) | 1553 (24,2) | 1398 (24,1) | 2520 (59,4) | |
|  | Urgent | 9026 (54,9) | 4302 (67,1) | 3352 (57,8) | 1372 (32,3) | |
|  | Very urgent | 1951 (11,9) | 549 (8,6) | 1051 (18,1) | 351 (8,3) | |
|  | Most urgent | 5 (0,0) | 3 (0,0) | 0 (0,0) | 2 (0,0) | |
|  |  |  |  |  |  | |
| 1. Falls | Total | 15503 (100) | 10009 (100) | 5494 (100) | 0 (0,0) | |
|  | Not urgent | 4751 (30,6) | 4260 (42,6) | 491 (8,9) | 0 (0,0) | |
|  | Urgent | 8967 (57,8) | 4887 (48,8) | 4080 (74,3) | 0 (0,0) | |
|  | Very urgent | 1751 (11,3) | 852 (8,5) | 899 (16,4) | 0 (0,0) | |
|  | Most urgent | 34 (0,2) | 10 (0,1) | 24 (0,4) | 0 (0,0) | |
| 1. Headache | Total | 15144 (100) | 9950 (100) | 2892 (100) | 2302 (100) | |
|  | Not urgent | 2218 (14,6) | 1075 (10,8) | 192 (6,6) | 951 (41,3) | |
|  | Urgent | 7820 (51,6) | 5123 (51,5) | 2015 (69,7) | 682 (29,6) | |
|  | Very urgent | 4642 (30,7) | 3558 (35,8) | 676 (23,4) | 408 (17,7) | |
|  | Most urgent | 464 (3,1) | 194 (1,9) | 9 (0,3) | 261 (11,3) | |
|  |  |  |  |  |  | |
| **Vital score** N (%) | Total vital score  Missing | 696518 (100)  0 (0,0) | 320406 (100)  0 (0,0) | 214267 (100)  0 (0,0) | 161845 (100)  0 (0,0) | |
| No vital signs | Total | 182022 (100) | 91576 (100) | 52775 (100) | 37671 (100) | |
| registered | Not urgent | 90418 (49,7) | 49654 (54,2) | 18237 (34,6) | 22527 (59,8) | |
|  | Urgent | 75094 (41,3) | 32135 (35,1) | 31438 (59,6) | 11521 (30,6) | |
|  | Very urgent | 13599 (7,5) | 8141 (8,9) | 2594 (4,9) | 2864 (7,6) | |
|  | Most urgent | 2911 (1,6) | 1646 (1,8) | 506 (1,0) | 759 (2,0) | |
| One or several vital | Total | 195237 (100) | 81533 (100) | 59819 (100) | 53885 (100) | |
| signs registered | Not urgent | 48007 (24,6) | 19487 (23,9) | 6836 (11,4) | 21684 (40,2) | |
|  | Urgent | 105633 (54,1) | 43965 (53,9) | 41922 (70,1) | 19746 (36,6) | |
|  | Very urgent | 36788 (18,8) | 16701 (20,5) | 10179 (17,0) | 9908 (18,4) | |
|  | Most urgent | 4809 (2,5) | 1380 (1,7) | 882 (1,5) | 2547 (4,7) | |
|  |  |  |  |  |  | |
| All vital signs | Total | 319529 (100) | 147297 (100) | 101673 (100) | 70289 (100) | |
| registered | Not urgent | 45788 (14,3) | 24315 (16,5) | 2645 (2,6) | 18828 (26,8) | |
|  | Urgent | 184168 (57,7) | 84277 (57,2) | 73929 (72,7) | 25962 (36,9) | |
|  | Very urgent | 81362 (25,5) | 36905 (25,1) | 24344 (23,9) | 20113 (28,6) | |
|  | Most urgent | 7941 (2,5) | 1800 (1,2) | 755 (0,7) | 5386 (7,7) |  |

**Legend:** Values are median [IQR, interquartile range] or absolute number (percentage). MTS: Manchester Triage System; ESI: Emergency Severity Index; NTS: Netherlands Triage Standard; ED: Emergency Department; LOS: Length of stay. Vital score: respiration rate, temperature, heart rate, blood pressure or oxygen saturation. Top ten presenting complaints: based on top ten presenting complaints of the entire database.
