# Supplementary material for: The association between urgency level and hospital admission, mortality and resource utilization in three emergency department triage systems: an observational multicenter study
Source: Scand J Trauma Resusc Emerg Med. 2025 May 1;33:72. doi: 10.1186/s13049-025-01392-5 (PMC12044865; doi:10.1186/s13049-025-01392-5)
Supplement: Supplementary file 4 — Additional File 4 Relative resource utilization [file 13049_2025_1392_MOESM4_ESM.docx]

**Additional File 4 Relative Resource Utilization**

Relative Resource Utilization in the Emergency Department

|  |  | **MTS** | **ESI** | **NTS** |
| --- | --- | --- | --- | --- |
| **Diagnostics** N (%) |  |  |  |  |
| Blood test | Not urgent | 31667 (33,9) | 3395 (12,2) | 29863 (47,4) |
|  | Urgent | 111659 (69,6) | 100619 (68,3) | 37816 (66,1) |
|  | Very urgent | 51524 (83,4) | 31290 (84,3) | 27119 (82,5) |
|  | Most urgent | 4213 (87,3) | 1892 (84,0) | 7840 (90,2) |
| Urine test (Sediment) | Not urgent | 7030 (7,5) | 790 (2,9) | 13311 (21,1) |
|  | Urgent | 31699 (19,8) | 32425 (22,0) | 14562 (25,4) |
|  | Very urgent | 13087 (21,2) | 12672 (34,1) | 8256 (25,1) |
|  | Most urgent | 1167 (24,2) | 188 (8,8) | 1825 (21,0) |
| Radiology (Conventional, | Not urgent | 48791 (52,2) | 8955 (32,3) | 28836 (45,7) |
| Ultrasound, CT) | Urgent | 95999 (59,9) | 88506 (60,1) | 34956 (61,1) |
|  | Very urgent | 40674 (65,9) | 29685 (80,0) | 20627 (62,7) |
|  | Most urgent | 3372 (69,9) | 1823 (85,1) | 6062 (69,7) |
| ECG | Not urgent | 10429 (11,2) | 1309 (4,7) | 9983 (15,8) |
|  | Urgent | 43813 (27,3) | 69788 (47,4) | 20579 (36,0) |
|  | Very urgent | 20386 (33,0) | 24787 (66,8) | 19042 (57,9) |
|  | Most urgent | 1630 (33,8) | 1183 (55,2) | 5924 (68,2) |
| **Interventions** N (%) |  |  |  |  |
| Fluid administered | Not urgent | 1844 (2,0) | 457 (1,6) | 8665 (13,7) |
|  | Urgent | 12006 (7,5) | 20885 (14,2) | 12020 (21,0) |
|  | Very urgent | 9674 (15,7) | 15454 (41,6) | 5935 (18,0) |
|  | Most urgent | 786 (16,3) | 1064 (49,7) | 1080 (12,4) |
|  |  |  |  |  |
| Medication administered | Not urgent | 8600 (9,2) | 6558 (23.7) | 17233 (27,3) |
|  | Urgent | 35976 (22,4) | 62994 (42.8) | 23038 (40,3) |
|  | Very urgent  Most urgent | 20898 (33,8) 1710 (35,4) | 25904 (69.8)  1077 (50,3) | 13704 (41,7)  4177 (48,1) |
| **Consultations** N (%) |  |  |  |  |
| 0 | Not urgent | 41745 (44,7) | 25913 (93,5) | 44664 (70,9) |
|  | Urgent | 58352 (36,4) | 126453 (85,9) | 28882 (50,5) |
|  | Very urgent | 22306 (36,1) | 27666 (74,5) | 10549 (32,1) |
|  | Most urgent | 1195 (24.8) | 887 (41,4) | 1581 (18,2) |
| 1 | Not urgent | 35207 (37,7) | 1683 (6,1) | 13519 (21,4) |
|  | Urgent | 68664 (42,8) | 18702 (12,7) | 18736 (32,7) |
|  | Very urgent | 22430 (36,3) | 8025 (21,6) | 14226 (43,3) |
|  | Most urgent | 1796 (37,2) | 837 (39,1) | 4051 (46,6) |
| ≥ 2 | Not urgent | 11965 (12,8) | 122 (0,4) | 4856 (7,7) |
|  | Urgent | 31381 (19,6) | 2134 (1,4) | 9611 (16,8) |
|  | Very urgent | 16698 (27,0) | 1426 (3,8) | 8110 (24,7) |
|  | Most urgent | 1810 (37,5) | 419 (19,6) | 3060 (35,2) |
|  |  |  |  |  |

**Legend:** Values are absolute numbers (percentage). Relative resource utilization is calculated as the number of resources used within a specific urgency level, divided by the total number of patients in that urgency level for the respective triage system (presented as percentages). MTS: Manchester Triage System; ESI: Emergency Severity Index; NTS: Netherlands Triage Standard; ED: Emergency Department; ECG: Electrocardiogram
