# Supplementary material for: The association between urgency level and hospital admission, mortality and resource utilization in three emergency department triage systems: an observational multicenter study
Source: Scand J Trauma Resusc Emerg Med. 2025 May 1;33:72. doi: 10.1186/s13049-025-01392-5 (PMC12044865; doi:10.1186/s13049-025-01392-5)
Supplement: Supplementary file 5 — Additional File 5 Odds ratios [file 13049_2025_1392_MOESM5_ESM.docx]

**Additional File 5: Odds Ratios for the association between urgency levels and in-hospital mortality and hospital admission by different triage systems**

**Table 1.** Odds Ratios for the association between urgency levels and in-hospital mortality by different triage systems

| **Urgency level** | **OR** | **Cohort OR** | **95%-CI** | **MTS**  **OR** | **95%-CI** | **ESI**  **OR** | **95%-CI** | **NTS**  **OR** | **95%-CI** | |
| --- | --- | --- | --- | --- | --- | --- | --- | --- | --- | --- |
| **Not urgent**  (Reference) |  |  |  |  |  |  |  |  |  | |
| **Urgent** | Crude | 2.86 | (2.65-3.08) | 3.10 | (2.77-3.48) | 8.90 | (6.27-12.64) | 2.97 | (2.65-3.33) | |
|  | Adjusted | 1.90 | (1.76-2.05) | 2.09 | (1.86-2.34) | 3.04 | (2.13-4.33) | 2.32 | (2.06-2.61) |  |
|  |  |  |  |  |  |  |  |  |  | |
| **Very urgent** | Crude | 9.72 | (9.01-10.47) | 10.12 | (9.04-11.33) | 47.96 | (33.81-68.04) | 5.19 | (4.63-5.82) | |
|  | Adjusted | 5.26 | (4.87-5.68) | 6.19 | (5.50-6.96) | 11.00 | (7.70-15.71) | 3.44 | (3.05-3.88) | |
|  |  |  |  |  |  |  |  |  |  | |
| **Most urgent** | Crude | 44.55 | (41.01-48.39) | 88.00 | (77.62-99.77) | 339.28 | (236.61-486.49) | 14.61 | (12.91-16.53) | |
|  | Adjusted | 29.59 | (27.11-32.30) | 65.37 | (57.03-74.92) | 170.16 | (117.40-246.64) | 10.70 | (9.36-12.24) | |

**Legend:** OR: Odds Ratio; AOR: Adjusted Odds Ratio; MTS: Manchester Triage System; ESI: Emergency Severity Index; NTS: Netherlands Triage Standard. AOR adjusted for age, sex, top ten presenting complaints and hospital type (academic/general). Reference group: not urgent. Top ten presenting complaints: 1. Extremity problems; 2. Feeling unwell; 3. Abdominal pain; 4. Dyspnea; 5. Chest pain; 6. Trauma major; 7. Wounds; 8. Urinary problems; 9. Falls; 10. Other (reference category).

**Table 2.** Odds Ratios for the association between urgency levels and hospital admission by different triage systems

| **Urgency level** | **OR** | **Cohort OR** | **95%-CI** | **MTS**  **OR** | **95%-CI** | **ESI**  **OR** | **95%-CI** | **NTS**  **OR** | **95%-CI** |
| --- | --- | --- | --- | --- | --- | --- | --- | --- | --- |
| **Not urgent**  (Reference) |  |  |  |  |  |  |  |  |  |
| **Urgent** | Crude | 2.60 | (2.57-2.64) | 3.23 | (3.17-3.29) | 6.55 | (6.24-6.87) | 2.44 | (2.39-2.50) |
|  | Adjusted | 1.98 | (1.95-2.01) | 2.47 | (2.42-2.52) | 3.47 | (3.29-3.65) | 2.36 | (2.30-2.43) |
|  |  |  |  |  |  |  |  |  |  |
| **Very urgent** | Crude | 6.86 | (6.75-6.97) | 6.83 | (6.67-6.99) | 27.77 | (26.38-29.23) | 4.32 | (4.20-4.44) |
|  | Adjusted | 4.92 | (4.83-5.01) | 5.70 | (5.55-5.86) | 12.55 | (11.87-13.26) | 3.85 | (3.72-3.98) |
| **Most urgent** |  |  |  |  |  |  |  |  |  |
|  | Crude | 17.13 | (16.39-17.90) | 34.24 | (30.91-37.93) | 179.11 | (149.55-214.52) | 8.02 | (7.61-8.46) |
|  | Adjusted | 14.98 | (14.29-15.70) | 35.99 | (32.35-40.03) | 134.89 | (111.87-162.65) | 8.73 | (8.22-9.27) |

**Legend:** OR: Odds Ratio; AOR: Adjusted Odds Ratio; MTS: Manchester Triage System; ESI: Emergency Severity Index; NTS: Netherlands Triage Standard. AOR adjusted for age, sex, top ten presenting complaints and hospital type (academic/general). Reference group: not urgent. Top ten presenting complaints: 1. Extremity problems; 2. Feeling unwell; 3. Abdominal pain; 4. Dyspnea; 5. Chest pain; 6. Trauma major; 7. Wounds; 8. Urinary problems; 9. Falls; 10. Other (reference category)
